# Agreement test of transcutaneous bilirubin and bilistick with serum bilirubin in preterm infants receiving phototherapy

**DOI:** 10.1186/s12887-018-1290-9

**Published:** 2018-09-29

**Authors:** Rinawati Rohsiswatmo, Hanifah Oswari, Radhian Amandito, Hikari Ambara Sjakti, Endang Windiastuti, Rosalina Dewi Roeslani, Indrayady Barchia

**Affiliations:** 1 Department of Child Health, Faculty of Medicine, Universitas Indonesia – Cipto Mangunkusumo Hospital, Jl Pangeran Diponegoro No. 71, Salemba, Kenari, Senen, Jakarta Pusat, DKI Jakarta 10430 Indonesia; 2 Neonatal Intensive Care Unit, Pondok Indah General Hospital, Jl Metro Duta Kav UE, Pondok Indah, Pondok Pinang, Kebayoran Lama, Jakarta Selatan, DKI Jakarta 12310 Indonesia

**Keywords:** Indonesia, Bilistick, Transcutaneous bilirubin, Phototherapy, Preterm infants

## Abstract

**Background:**

This study compares the minimally invasive Bilistick and a noninvasive method with standard Total Serum Bilirubin (TSB) measurement in preterm newborns receiving phototherapy. We assess the agreement of Transcutaneous Bilirubinometer (TcB) and Bilistick bilirubin measurements with standard TSB measurement in preterm infants receiving phototherapy.

**Methods:**

Bilirubin was measured by using TcB and Bilistick in 94 preterm infants in RSCM Jakarta Neonatal Ward from October 2016 to March 2017, with gestational ages of < 35 weeks, before phototherapy and after 24 and 48 h of phototherapy.

**Results:**

There was significant correlation before, at 24 and 48 h of phototherapy between TSB and either TcB (*r* = 0.874; *r* = 0.889; *r* = 0.878 respectively; *p* < 0.0001), or Bilistick (*r* = 0.868; *r* = 0.877; *r* = 0.918 respectively; *p* < 0.0001). The mean difference and limits of agreement before, at 24 and 48 h of phototherapy between TcB and TSB were 0.81 ± 1.51 mg/dL (− 2.14 to 3.77 mg/dL); 0.43 ± 1.57 mg/dL (− 2.66 to 3.51 mg/dL); 0.41 ± 1.58 mg/dL (− 2.69 to 3.50 mg/dL), respectively. For Bilistick they were − 1.50 ± 1.47 mg/dL (− 4.38 to 1.38 mg/dL); − 1.43 ± 1.47 mg/dL (− 4.32 to 1.46 mg/dL); − 1,15 ± 1.31 mg/dL (− 3,72 to 1,42 mg/dL), respectively.

**Conclusions:**

Both methods are reliable for measuring TSB before, during, and after phototherapy in preterm infants. TcB tends to overestimate while Bilistick underestimates TSB.

## Background

Hyperbilirubinemia is one of the most common problems arising in the neonatal period. Hyperbilirubinemia in neonates often develops in the first week of life, ranging in frequency from 60% in term and 80% in preterm infants [1–3]. Phototherapy is still the primary treatment to prevent further complications for newborns with hyperbilirubinemia, especially for premature infants who have a higher risk of bilirubin encephalopathy [4]. During the course of phototherapy, it is necessary to monitor the bilirubin levels periodically until phototherapy is completed in order to prevent overtreatment. However, regular blood taking can cause problems such as anemia and increased risk of infection in newborns with hyperbilirubinemia. The risk increases especially in preterm infants with lower blood volume and altered immune status. Serum bilirubin measurement is a gold standard for measuring total serum bilirubin (TSB) levels for both detection and evaluation during phototherapy. However, this measurement is an invasive procedure that poses a higher risk of infection, pain, and requires a rather large amount of blood [5–7]. Currently, there are several alternatives for measurement of bilirubin levels. In this study, we will be focusing on the Bilistick System. Compared to TSB, which requires large volumes of blood, the Bilistick System requires only 25 μL of capillary blood. With regards to its cost, the Bilistick System is considerably cheaper than the non-invasive transcutaneous bilirubinometer (TcB); €1300 for transcutaneous bilirubin (JM 103) compared to €600 for Bilistick device (2016 Version) and €1.5 for each test strip and transfer pipette.

Several studies on transcutaneous and Bilistick bilirubin measurements have been conducted to assess the validity of both devices. The correlation between TcB and TSB results in previous studies was strong (*r* = 0.835; *p* < 0.0001), but this study did not measure bilirubin levels during phototherapy [8]. A previous meta-analysis study comparing the measurement of TcB and TSB levels during phototherapy obtained *r* = 0.64 (95% CI 0.43–0.77) on the measurement of transcutaneous bilirubin in the sternal region, but this study was performed on near-term and term infants [9].

Studies comparing Bilistick and serum bilirubin showed a strong correlation (*r* = 0.961 and *r* = 0.914; *P* < 0.0001) but they did not include infants receiving phototherapy [10]. A study comparing bilirubin measurement using transcutaneous bilirubin and Bilistick has been recently reported, with a similar limit of agreement of the Bilistick System (− 5.8 to 3.3 mg/dL) and JM-103 system (− 5.4 to 6.0 mg/dL) versus the clinical laboratory; however, this study only involved term infants and not those being treated by phototherapy [11].

To our knowledge, no previous study has been conducted comparing the measurement of TcB and Bilistick with total serum bilirubin during phototherapy in preterm infants. The aim of this study was to assess the agreement of TcB and Bilistick bilirubin measurements in hyperbilirubinemic preterm infants before and during phototherapy in the hope of determining the best alternative method for measurement of bilirubin levels to reduce the risk of anemia and infections in preterm infants. This is especially important for places with limited laboratory facilities that require measurement systems that are accurate, easy to use, inexpensive, and provide fast results.

## Methods

### Study population

This study was conducted in the Neonatology Division of the Department of Child Health Faculty of Medicine, Universitas Indonesia – Cipto Mangunkusumo Hospital (RSCM) Jakarta. The inclusion criteria were: hyperbilirubinemic preterm infants age ≤ 14 weeks, gestational age < 35 weeks old who are receiving phototherapy in the Neonatology Division of RSCM Jakarta and with signed parental consent for inclusion in the study. Exclusion criteria were: preterm infants with a prior history of phototherapy or previous exchange transfusion and all preterm infants with defects at the site of measurement. Decision to initiate phototherapy was based on the Indonesian Pediatric Society (IDAI) guidelines for preterm infants with hyperbilirubinemia [12]. Bilirubin measurement was conducted only for the first 48 h of phototherapy after which the neonate received standard therapy including continued phototherapy if indicated.

### Laboratory investigations

For each participating hyperbilirubinemic newborn, a thorough explanation of the research was given to the parents and parental consent to participate in the study was obtained. Identification of the patient included: name, age, gender, parent’s name and relationship with parents, gestational age, and history of phototherapy. Ballard score was calculated, then the weight was measured using baby scales (20 kg with 0.1 kg precision) (Seca, Germany), and body length and head circumference were measured using an infantometer and measuring tape (100 cm length with 0.1 cm precision) (Seca, Germany). For each infant, bilirubin was measured using TcB, Bilistick and total serum bilirubin lab measurement. The TcB measurement was performed 3 times in a row with Dragger JM 103 in the midsternum area, and the average of the 3 measurements was obtained. All measurements were made by a trained nurse or physician. For the Bilistick measurement, 25uL of capillary blood was collected by a nurse, and then applied to a test strip and inserted in the Bilistick reader. The TSB concentration is determined by reflectance spectroscopy within 3 min of loading.

The TSB measurement was performed by taking 0.6 ml of venous blood, putting it into a vacuum tube, and sending it to Cipto Mangunkusumo Hospital Clinical pathology laboratory in Jakarta, where they employed the chemical oxidation method utilizing vanadate as the oxidizing agent using the ADVIA Chemistry Total Bilirubin 2 device (Siemens, Germany).

### Statistical analysis

Categorical data are presented in the form of frequency distribution, proportion, and percentage, while continuous-scale data are presented as mean and standard deviation or median and range. The correlation between the gold standard measurement and the tool under test was calculated using Pearson correlation (normal distribution) and Spearman correlation (abnormal distribution). Bland-Altman test was used to calculate the agreement of the mean difference between the gold standard measurement and the tested tool. The mean difference is expressed as mean ± SD, while the limit of agreement is calculated based on the mean ± 1.96 SD. Statistical Package for the Social Sciences (SPSS) version 18.0 for Windows was used for all statistical analyses. This research was approved by the Medical Research Ethics Committee of the Faculty of Medicine, University of Indonesia and the licensing of research sites from Cipto Mangunkusumo Hospital.

## Results

During the study period, there were 120 preterm infants less than 35 weeks of gestation who suffered hyperbilirubinemia and 96 of them (80%) had indications for phototherapy. Of these 96 infants, 2 infants were not treated with phototherapy due to clinical deterioration and eventual death. The remaining 94 infants were enrolled in the study.

The characteristics of 94 participants included in the study are reported in Table 1. There were 53 (56.4%) males and 41 (43.6%) females. The mean age of the subjects at the time of hyperbilirubinemia diagnosis was 2.9 days with a range of 1 to 10 days. The mean of gestational age of subjects was 31.3 weeks with a range of 26 to 34 weeks while mean weight of subjects was 1466.73 g with a range of 700 to 2450 g.

**Table 1:** Characteristics of subjects (*n* = 94)

Characteristics	
Gender	
Male	53 (56.4%)
Female	41 (43.6%)
Age (days) Mean ± SD (range)	2.94 ± 1.66 (1 to 10)
Gestational age (weeks) Mean ± SD (range)	31.27 ± 2.32 (26 to 34)
Weight (g) Mean ± SD (range)	1466.73 ± 442.24 (700 to 2450)

Profiles of total serum bilirubin, transcutaneous bilirubin, and Bilistick levels before phototherapy, and after 24 h and 48 h of phototherapy are reported in Table 2.

**Table 2:** Profile of bilirubin levels

Measurement	Bilirubin level
Before phototherapy	
Total serum bilirubin (mg/dL) Mean ± SD (range)	10.18 ± 2.91 (4.43 to 19.70)
Transcutaneous (mg/dL) Mean ± SD (range)	10.99 ± 3.07 (4.70 to 19.60)
Bilistick (mg/dL) Mean ± SD (range)	8.68 ± 2.78 (4.20 to 14.80)
24 h of phototherapy	
Total serum bilirubin (mg/dL) Mean ± SD (range)	9.75 ± 3.04 (3.25 to 18.20)
Transcutaneous (mg/dL) Mean ± SD (range)	10.18 ± 3.44 (1.90 to 19.70)
Bilistick (mg/dL) Mean ± SD (range)	8.33 ± 2.86 (3.20 to 17.90)
48 h of phototherapy	
Total serum bilirubin (mg/dL) Median ± SD (range)	8 (0.58 to 21.26)
Transcutaneous (mg/dL) Median ± SD (range)	7.9 (1.70 to 22.30)
Bilistick (mg/dL) Median ± SD (range)	6.85 (1.20 to 17.20)

Kolmogorov-Smirnov and Shapiro-Wilk test were used to test the normality of data, from which we obtained a normal distribution with *p* > 0.05 for bilirubin measurement data before phototherapy and after 24 h of phototherapy. While for measurement of 48-h bilirubin phototherapy, we obtained an abnormal distribution with a value of *p* < 0.05.

**Fig. 1:**
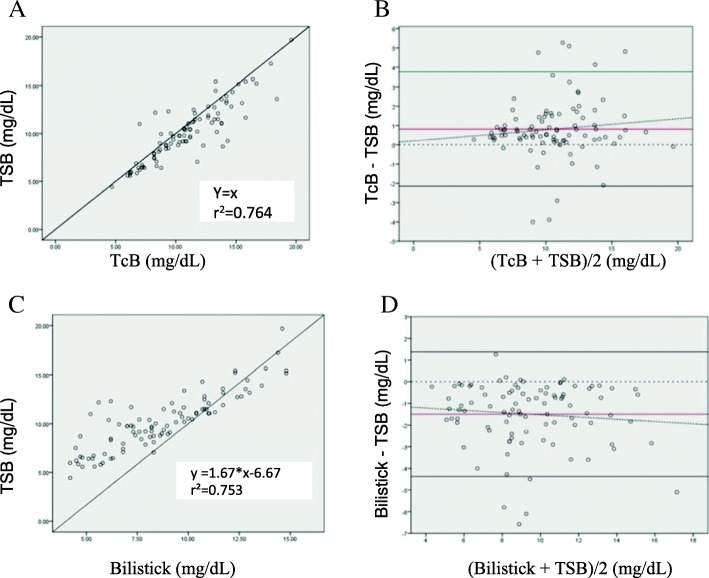
Scatter and Bland Altman plot between TcB and TSB (**a** and **b**), Bilistick and TSB (**c** and **d**) before phototherapy, Y equation = linear equation obtained from linear regression analysis. Y = estimated TSB, X = Bilirubin level of the device being tested () = average difference () = agreement limit () = tendency of average difference () = 0 point, standard to observe distance with red line

The Pearson correlation test of total serum bilirubin level and transcutaneous bilirubin before phototherapy showed a significant positive correlation between total serum bilirubin and transcutaneous bilirubin (Fig. 1a) (*r*^2^ = 0,764, *p* < 0.0001).

Transcutaneous bilirubin levels tended to overestimate total serum bilirubin before phototherapy with a mean difference of 0.81 mg/dL (SD 1.51) with a 95% CI of 0.50 to 1.12 and the limits of agreement were − 2.14 and 3.77 mg/dL. The Bland-Altman plot shows that the higher the bilirubin level, the wider the difference between the 2 methods (Fig. 1b).

As shown in Fig. 1c there was a significant and positive correlation between total serum bilirubin and Bilistick bilirubin measurement before phototherapy (*r*^2^ = 0.753, *p* < 0.0001).

Bilistick tended to underestimate total serum bilirubin before phototherapy measurement with a mean difference of − 1.50 mg/dL (SD 1.47) with a 95% CI of − 1.80 to − 1.20 with the limits of agreement of − 4.38 and 1.38 mg/dL. The Bland-Altman plot shows the higher the bilirubin level, the wider the difference between Bilistick and total serum bilirubin measurement (Fig. 1d).

**Fig. 2:**
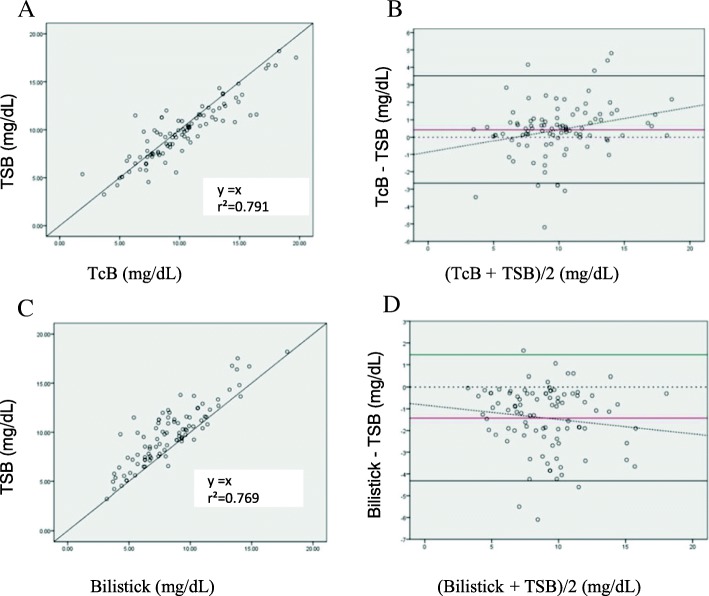
Scatter and Bland Altman plots between TcB and TSB (**a** and **b**), Bilistick and TSB (**c** and **d**) at 24 h of phototherapy. Y equation = linear equation obtained from linear regression analysis. Y = estimated TSB, X = Bilirubin level of the device being tested () = average difference () = agreement limit () = tendency of average difference () = 0 point, standard to observe distance with red line

After 24 h of phototherapy, we found a significant positive correlation between total serum bilirubin and transcutaneous bilirubin (*r*^2^ = 0.791, *p* < 0.0001 (Fig. 2a). TcB overestimates total serum bilirubin with a mean difference of 0.43 mg/dL (SD 1.57) with 95% CI of 0.10 to 0.75 and limits of agreement of − 2.66 and 3.51 mg/dL (Fig. 2b). There was a positive and significant correlation between total serum bilirubin and Bilistick bilirubin (*r*^2^ = 0.769, *p* < 0.0001) (Fig. 2c). Bilistick underestimates total serum bilirubin with a mean difference − 1.43 mg/dL (SD 1.47) with 95% CI -1.73 to − 1.13 and the limits of agreement were − 4.32 and 1.46 mg/dL. The Bland-Altman plot shows the higher the bilirubin level, the wider the difference between Bilistick and total serum bilirubin measurement (Fig. 2d).

**Fig. 3:**
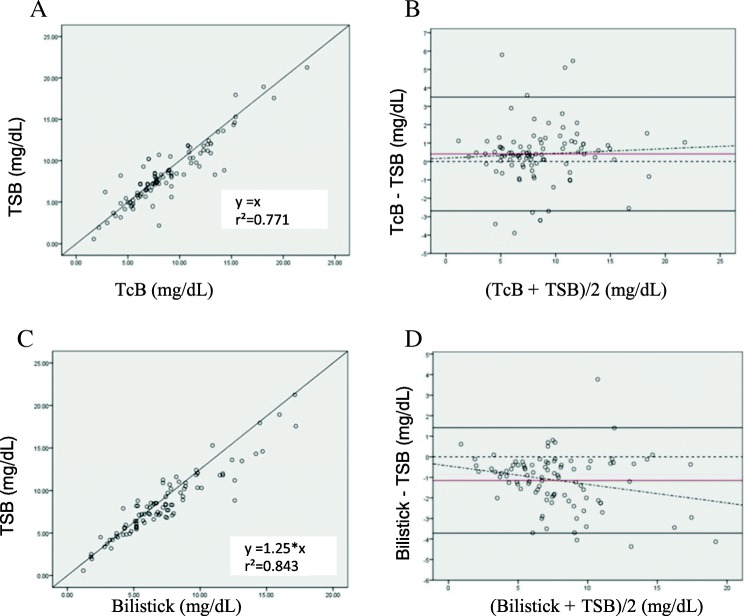
Scatter and Bland Altman plot between TcB and TSB (**a** and **b**), Bilistick and TSB (**c** and **d**) at 48 h of phototherapy. Y equation = linear equation obtained from linear regression analysis. Y = estimated TSB, X = Bilirubin level of the device being tested () = average difference () = agreement limit () = tendency of average difference () = 0 point, standard to observe distance with red line

The same pattern was observed after 48 h of phototherapy. A significant and positive correlation was present between total serum bilirubin and transcutaneous bilirubin (*r*^2^ = 0.771, *p* < 0.0001) (Fig. 3a); TcB overestimates total serum bilirubin with a mean difference of 0.41 mg/dL (SD 1.58) with 95% CI 0.08 to 0.73 and the limits of agreement were − 2.69 and 3.50 mg/dL. The Bland-Altman plot shows that the higher the bilirubin level the wider the difference between transcutaneous bilirubin level and total serum bilirubin (Fig. 3b). A significant and positive correlation was also found between total serum bilirubin and Bilistick (*r*^2^ = 0.843, *p* < 0.0001) (Fig. 3c). Bilistick bilirubin tends to underestimate total serum bilirubin with a mean difference of − 1.15 mg/dL (SD 1.31) with 95% CI -1.42 to − 0.88 and the limits of agreement were − 3.72 and 1.42 mg/dL. The Bland-Altman plot shows, the higher the bilirubin level, the wider the difference between Bilistick bilirubin level and total serum bilirubin (Fig. 3d).

## Discussion

This study assessed the correlation and agreement of TcB and Bilistick bilirubin measurement with TSB before and during phototherapy in preterm infants of gestational age less than 35 weeks. There was a very strong and significant positive correlation between TcB and Bilistick bilirubin measurement with TSB before and after 24 or 48 h of phototherapy. The results we obtained were similar to the meta-analysis of TcB measurement in preterm infants [9]. Prior to phototherapy, JM 103 had a strong correlation of *r* = 0.87 (95% CI of 0.82–0.91), and an even stronger correlation with preterm infants of less than 32 weeks (*r* = 0.89 (95% CI of 0.82–0.93)) [13]. Other studies obtained even stronger correlations in preterm infants with a gestational age of less than 28 weeks (*r* = 0.92 and *r* = 0.94, [14, 15]). The result of correlation on 24-h phototherapy TcB measurement in our study was superior to previous studies in near term and term infants [9]. We assume this difference is due to our participants being premature infants with gestational ages of less than 35 weeks, as their thinner skin thickness may improve light absorption and reflection from the subcutaneous tissue to the appliance. The accuracy of Bilistick was also comparable with previous studies in term infants who did not receive phototherapy, suggesting that Bilistick may be reliably used in preterm infants before and after phototherapy [10, 11, 13–16].

Measurement of TcB before phototherapy and after 24 and 48 h of phototherapy tended to overestimate by 0.81, 0.43, and 0.41 mg/dL compared with TSB. These results did not greatly differ from previous studies [11, 13, 17, 18]. TcB measurement before phototherapy and after 24 h and 48 h of phototherapy overestimated bilirubin level with a mean difference of less than 1 mg/dL and showed very strong positive correlation. This led to the conclusion that TcB bilirubin measurement was reliable in preterm infants both before and during phototherapy. Why the TcB measurement tended to overestimate serum bilirubin is not clear, but we hypothesized that it is related to an increased blood flow in the skin, which is inversely proportional to weight gain and gestational age. This is revealed in our study of preterm infants with gestational ages of less than 35 weeks who have high blood flow and low skin thickness. Phototherapy increases blood flow in the skin by increasing the body temperature. An increase in body temperature of 0.5–1 °C leads to an increase of 3 times the blood flow in the skin [19]. In addition, some TcB devices while usable up to a bilirubin level of 20 mg/dL, the reliability of the result is questionable at levels above 15 mg/dL.

Bilistick measurement before phototherapy, and after 24 h and 48 h of phototherapy tended to underestimate by 1.5, 1.43, and 1.15 mg/dL compared with TSB. Bilistick measurement appears to be lower with a mean difference of less than 1.5 mg/dL. This result is similar with what was reported in term infants who did not receive phototherapy [11]. Since this is the first study performed in preterms using the Bilistick system, additional data needs to be collected to verify that this small underestimation is correct.

Based on the tendency of both methods to overestimate or underestimate, the Indonesian guideline for phototherapy or exchange transfusion might require some changes in cases where doctors decide to use the Bilistick alone. This underestimation would have led to 11 neonates not receiving phototherapy when phototherapy would have been indicated by traditional serum bilirubin measurements in our population.

### Limitation

We did not use the newest version of Bilistick because it was not available during our time of study [11]. The population of our study includes mostly patients of Sumatran and Javanese descent, in whom skin tones do not contrast greatly enough between light and dark skin tone for transcutaneous bilirubin results to show any of the discrepancies reported in Caucasian and African-American races [20–22]. We also did not have enough samples to statistically analyze the difference between extreme premature and premature babies.

## Conclusions

Both TcB and Bilistick show equal reliability and can be used as an alternative measurement methods for monitoring bilirubin levels in term and preterm newborns, as well as before and after phototherapy. However, TcB tends to overestimate TSB while Bilistick underestimates TSB.

## Abbreviations

CI: Confidence Interval
RSCM: Cipto Mangunkusumo Hospital
SD: Standard deviation
SPSS: Statistical package for the social sciences
TcB: Transcutaneous bilirubin
TSB: Total serum bilirubin
